# Stem Cell Factor Neutralization Protects From Severe Anaphylaxis in a Murine Model of Food Allergy

**DOI:** 10.3389/fimmu.2021.604192

**Published:** 2021-03-09

**Authors:** Catherine Ptaschinski, Andrew J. Rasky, Wendy Fonseca, Nicholas W. Lukacs

**Affiliations:** ^1^Department of Pathology, Ann Arbor, MI, United States; ^2^Mary H. Weiser Food Allergy Center, University of Michigan, Ann Arbor, MI, United States

**Keywords:** food allergy, stem cell factor, mast cell, anaphylaxis, innate lymphoid cell

## Abstract

Food allergy is a growing public health problem with ~15 million people affected in the United States. In allergic food disease, IgE on mast cells bind to ingested antigens leading to the activation and degranulation of mast cells. Stem cell factor (SCF) is mast cell growth and activation factor that is required for peripheral tissue mast cells. We targeted a specific isoform of SCF, the larger 248 amino acid form, that drives peripheral tissue mast cell differentiation using a specific monoclonal antibody in a model of food allergy. Ovalbumin sensitized and intragastrically challenged mice were monitored for symptoms of anaphylaxis including respiratory distress, diarrhea, and a reduction in body temperature. During the second week of challenges, allergic mice were injected with an antibody to block SCF248 or given IgG control. Mice treated with α-SCF248 had a decreased incidence of diarrhea and no reduction in body temperature suggesting a reduction in anaphylaxis compared to IgG control treated animals. Re-stimulated mesenteric lymph nodes indicated that α-SCF248 treated mice had decreased OVA-specific Th2 cytokine production compared to IgG control treated allergic animals. The reduction of food induced anaphylaxis was accompanied by a significant reduction in gut leak. The mesenteric lymph node cells were analyzed by flow cytometry and showed a decrease in the number of type 2 innate lymphoid cells in mice injected with α-SCF248. Morphometric enumeration of esterase+ mast cells demonstrated a significant reduction throughout the small intestine. Using a more chronic model of persistent food-induced anaphylaxis, short term therapeutic treatment with α-SCF248 during established disease effectively blocked food induced anaphylaxis. Together, these data suggest that therapeutically blocking SCF248 in food allergic animals can reduce the severity of food allergy by reducing mast cell mediated disease activation.

## Introduction

The incidence and severity of food allergy early in life has been growing considerably over the past two decades. Presently, it is estimated that one in 13 children have food allergic responses that predispose them to anaphylaxis (1, 2). Diagnostic assessment of children with potential food anaphylaxis include elevated food specific serum IgE and severe skin challenge reactivity (3). Unfortunately, these latter parameters are not predictive of whether a child will fail a food challenge in the clinic (4). Furthermore, it is unclear whether a negative food challenge is predictive of future potential reactivity to accidental challenge later in life. Importantly, we do know that the mechanisms that drive an anaphylactic response begins with a rapid and systemic activation of mast cells that cause the release of mediators that initiate the vascular response (5, 6). Several strategies have been studied and utilized in the clinics with some specifically blocking mast cell activation, especially targeting IgE (7–9). Recent use of biologics primarily targeting type 2 immune responses have been suggested or are beginning in initial trials, including α-IL-4/13R, α-IL-5, and α-IL-33 (10–14). These latter therapeutic targets are focused on the type 2 immune response that inhibit immune environments but do not alter the effector responses of anaphylaxis directly. Few strategies have pursued reduction of mast cell numbers as a means for inhibiting adverse allergic responses. The presence of increased mast cell numbers in mucosal gastrointestinal (GI) tract tissue may be critical for driving the severity of anaphylactic responses in patients with increased food specific IgE.

A key molecule that has a central role in mast cell development, survival and activation is stem cell factor (SCF also knowns as kit ligand) (15, 16). In both humans and mice, endogenous SCF occurs in two isoforms, “membrane” (220 amino acids) and “soluble” (248 amino acids) forms (17, 18). They differ by the inclusion of Exon 6 in the SCF248 form, both are membrane expressed, and can induce a c-kit receptor-dependent signal to cells by enhanced cross-linking. SCF248 is known as the “soluble” form because it is enzyme cleavable within exon 6 that allows the SCF extracellular domain fragment to be more easily released from the surface of the cell. This is the source of the vast majority of serum SCF248, which is monomeric and therefore cannot activate c-kit^+^ cells due to an inability to cross-link c-kit (19). Differences in the isoforms' biology come from studies with Sl/Sld mice that are runted, anemic (due to SCF220's role in erythropoiesis), and have altered inflammatory responses. Sl/Sld mutant mice were transfected with either SCF248 or SCF220 and examined to determine whether the specific forms could differentially reconstitute defective biology. Induced SCF220 expression corrected the runting and anemia with little effect on the inflammatory responses in the Sl/Sld mice, whereas SCF248 expression did not correct the runting or anemia but increased myeloid cell populations including mast cells (20). A separate study that utilized mice that express only SCF220 showed that animals developed normally with no runting or anemia, yet did not have peripheral mast cells (21). Together, these genetic studies define that these SCF isoforms have different biologic functions with SCF220 associated with homeostatic and SCF248 associated with peripheral immune responses, especially mast cells. We have recently published that SCF248 is the dominate form expressed in peripheral tissues during chronic disease. Furthermore, our studies indicate that α-SCF248 specific mAb can inhibit chronic asthma and severe remodeling diseases by reducing pathogenesis, cytokine production and critical c-kit^+^ innate cells, including mast cells, eosinophils and ILC2s (22, 23).

In the present set of studies we have identified that specifically blocking SCF248 during and after food allergen induced disease can block anaphylaxis and is associated with diminished numbers of mast cells throughout the small intestine. Furthermore, inhibition of SCF248 reduced the overall type 2 immune response and reduced serum IgE suggesting that the systemic immune environment may be affected using this strategy. Importantly, the blockade of SCF248 led to reduced gut leak that suggests a more stable gut barrier that is necessary for controlling systemic allergen distribution. Our studies highlight that blocking a specific isoform of SCF (SCF248) associated with development and activation of mast cells diminished the anaphylactic phenotype in food allergic mice that may be developed for future therapeutic application in food-induced anaphylaxis.

## Materials and Methods

### Animals

Female BALB/c mice were purchased from Jackson Laboratories and used at 6–7 weeks of age. All experiments were approved by the University of Michigan Institutional Animal Care and Use Committee.

### Allergic Model and Monoclonal Antibody Production

**Figure 1:**
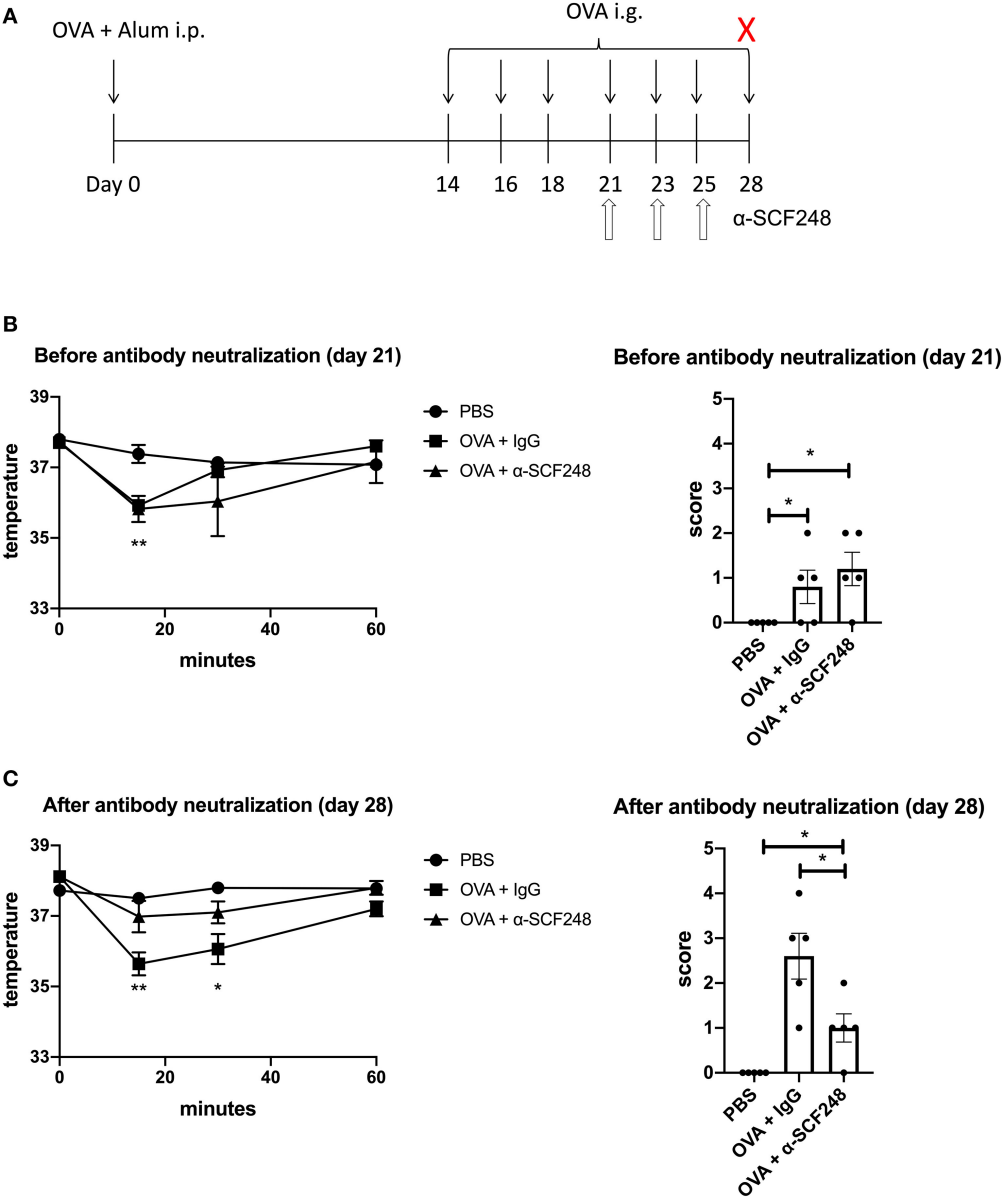
Neutralizing SCF248 reduces anaphylactic symptoms in allergic mice. **(A)** Model to induce anaphylaxis. Mice were sensitized with OVA and alum on day 0, then challenged with OVA by oral gavage on days 14, 16, 18, 21, 23, 25, and 28. SCF248 was neutralized by intraperitoneal injection on days 21, 23, and 25. Animals were euthanized 60 min after final challenge. **(B)** Prior to SCF248 neutralization (day 21, challenge four), temperatures were recorded at 15, 30, and 60 min following oral challenge. Symptoms were continuously monitored for 60 min, and mice were assigned as score on a scale of one to five based on symptom presentation. *p < 0.05, **p < 0.01 for OVA + IgG and OVA + αSCF248 compared to PBS. **(C)** Following SCF248 neutralization (day 28, challenge seven), temperatures were monitored for 60 min, and animals were euthanized. Mice were assigned a clinical score based on symptoms. Results are from five mice per group. Date show mean ± SEM, and are from one of three independent experiments. *p < 0.05, **p < 0.01 for OVA + IgG compared to PBS and OVA + αSCF248.

Mice were treated as in Figure 1A or **Figure 5A**. Mice were sensitized intraperitoneally on day 0 with 100 μg of endotoxin-free ovalbumin (OVA, Invivogen, San Diego, CA, USA) adsorbed to 1mg of alum (Imject® Alum, ThermoFisher Scientific, Waltham, MA, USA), then were challenged with 5 mg of OVA protein (Sigma Cat#A5503, St. Louis, MO, USA) in 200 μl or with 200 μl of PBS on days 14, 16, 18, 21, 23, 25, and 28. Mice were fasted for 5–6 h prior to each challenge. To neutralize SCF248, mice were injected with 20 mg/kg of α-SCF248 or control IgG on days 21, 23, and 25, or on days 28, 31, and 35 for the delayed response model. The specific SCF248 monoclonal antibody was made as previously described (22). A similarly produced control IgG1 antibody was used to a non-mammalian target (Genscript).

After each challenge, animals were monitored for 60 min, and rectal temperatures were recorded at 0, 15, 30, and 60 min following oral gavage with PBS or OVA. A score of 0–5 was assigned to each mouse based on anaphylaxis symptoms, as previously described (24). The scale is as follows: (0) no symptoms; (1) scratching and rubbing around the nose and head; (2) puffiness around the eyes and mouth, diarrhea, pilar erecti, reduced activity, and/or decreased activity with increased respiratory rate; (3) wheezing, labored respiration, and cyanosis around the mouth and the tail; (4) no activity after prodding or tremor and convulsion; (5) death.

### Histopathology

The small intestine was removed and flushed with cold PBS. Tissue was divided into duodenum, jejunum, and ileum. Each section was opened longitudinally and coiled onto a wooden stick to create a roll. Tissue was fixed in 10% formalin, followed by 70% ethanol, then embedded in paraffin and 5 μm sections were cut and mounted onto slides. Mast cells were visualized using chloroacetate esterase staining as previously described, as this staining protocol is known to detect mucosal mast cells (25, 26). At least five high powered fields (HPF) were counted on each section of the intestine per animal.

### Flow Cytometry

Mesenteric lymph nodes were removed and single cells were isolated by enzymatic digestion with 1 mg/ml collagenase A (Roche, Indianapolis, IN, USA) and 20 U/ml DNaseI (Sigma, St. Louis, MO, USA) in RPMI containing 10% FCS. Lymphocytes from the lamina propria were isolated as previously described (27). Briefly, the small intestine was opened longitudinally and mucus was removed by washing the tissue with PBS + 2% FCS + 5mM DTT at 37°C for 20 min. The intestinal epithelial cell lining was removed by three washes with PBS + 2% FCS% 5 mM EDTA, each for 10 min at 37°C. The tissue was then minced into small pieces, and at digested in HBSS supplemented with 10mM HEPES, 37.5 U/ml Liberase TM (Roche Applied Science, Indianapolis, IN, USA), and 300 U/ml DNase I for 30 min at 37°C. The resulting cell suspension was filtered through a 70 μm sieve, counted, and plated for flow cytometry at 2 × 10^6^ per well. Cells were washed and resuspended in PBS and live cells were identified using LIVE/DEAD Fixable Yellow Dead Cell Stain kit (ThermoFisher Scientific, Waltham, MA, USA), then were washed and resuspended in PBS with 1% FCS and Fc receptors were blocked with purified α-CD16/32 (clone 93; BioLegend, San Diego, CA, USA). Surface markers were identified using antibodies against the following antigens, all from BioLegend: CD3 (17A2), CD11b (M1/70), CD45R/B220 (RA3-6B2), Ter119 (TER-119), Gr-1 (RB6-8C5), CD45 (30-F11), SiglecF (E50-2440), c-kit/CD117 (2B8), CD90 (5E10), IL7Rα/CD127 (A019D5), ST2/IL33Rα (DIH4), and FcεRIα (MAR-1). Intracellular staining for GATA3 (clone 16E10A23 was performed using the Transcription Factor Staining Buffer Set (eBioscience) according to the manufacturer's protocol. Eosinophils were identified as CD45^+^SiglecF^+^SSC^hi^. Mast cells were identified as CD45^+^c-kit^+^FcεRIα^+^. ILC2s were identified as lin^−^CD45^+^c-kit^+^CD90^+^IL7Rα^+^ST2^+^Gata3^+^. Lineage negative cells were defined as CD3^−^CD11b^−^CD45R/B220^−^Ter119^−^Gr1^−^.

### Lymph Node Restimulation

Mesenteric lymph nodes (MLN) were removed and single cells were isolated by enzymatic digestion, as described above. 5 × 10^5^ cells were plated in 200 μl of complete medium (RPMI 1640 supplemented with 10% FCS, L-glutamine, penicillin/streptomycin, non-essential amino acids, sodium pyruvate, 2-mercaptoethanol) and were restimulated with 50 μg/ml of ovalbumin for 48 h. Unstimulated cells were used as baseline controls. Supernatants were collected and levels of the cytokines IL-4, IL-5, and IL-13 were measured by Bioplex assay (Bio-Rad, Hercules, CA, USA).

### IgE and mMCP1 Enzyme Linked Immunosorbant Assay

OVA-specific antibody isotypes were measured in the serum of treated mice. Immunosorbant 96-well plates (ThermoFisher Scientific) were coated with OVA overnight at 4°C, then incubated with blocking buffer (1% dry non-fat milk in PBS) for 1 h at 37°C. Serial dilutions of serum were made in 1% BSA and were incubated on the plates overnight at 4°C. Plates were washed PBS + 0.05% Tween 20. Alkaline phosphatase-labeled secondary antibodies (Jackson ImmunoResearch, West Grove, PA, USA) were incubated at 37°C for 1.5 h. Plates were washed and incubated at room temperature with p-nitrophenyl phosphate (Sigma-Aldrich, St. Louis, MO, USA) and color was developed for 1 h. Plates were read on a SpectraMax iD3 (Molecular Devices, San Jose, CA, USA) at 405 nm, and results are presented as optical density. Optical density in allergic mice was calculated as OD_allergic_–(OD_control_ + 2^*^SD_control_). mMCP1 was measured in the serum using a mouse MCPT-1 kit (Invitrogen) according to the manufacturer's instructions.

### Epithelial Barrier Permeability Assay

Mice were fasted for 4 h, then given 150 μl of 4 kDa FITC-dextran by oral gavage at a concentration of 80 mg/ml. Blood was collected after 4 h and centrifuged to separate plasma. Plasma was then diluted 1:10 in PBS at and read on a SpectraMax iD3 at 530 nm with excitation at 485 nm. Serial dilutions of FITC-dextran were made to create a standard curve.

### Western Blot of JAM-A

Protein was extracted from a section of the jejunum by homogenization in RIPA buffer (Cell Signaling Technology, Danvers, MA, USA) supplemented with 1mM PMSF (ThermoFisher Scientific). Protein was clarified by centrifugation, and concentration was determined by Bradford assay as previously described (28). Fifteen μg of protein was loaded onto Bis-Tris polyacrylamide gels (Invitrogen), and protein was transferred onto a nitrocellulose membrane using an iBlot 2 dry blotting system (ThermoFisher Scientific). Western blot was performed using antibodies again JAM-A (R&D Systems, Minneapolis, MN, USA) and b-actin (Cell Signaling Technology), followed by horseradish peroxidase-conjugated secondary antibodies. JAM-A was detected, followed by stripping of the membrane with Restore™ Western Blot Stripping Buffer (ThermoFisher Scientific), then detection of b-actin. Detection was done using enhanced chemiluminescence (GE Healthcare, Pittsburgh, PA, USA). Band densities were measured using the rectangle measurement tool on ImageLab software (BioRad, Hercules, CA, USA), and JAM-A densities were normalized to b-actin.

### Statistical Analysis

Results are expressed as mean ± standard error. Statistical significance was measured by one-way or two-way ANOVA followed by *post-hoc* Student's *t*-test as appropriate. A *p* ≤ 0.05 was considered significant.

## Results

### Neutralization of SCF248 Protects Mice From Food-Induced Anaphylaxis With Reduction in Intestinal Mast Cells

The induction of food allergy in mice has been established using several methodologies. In these studies we utilized systemic immunization with alum and ovalbumin to establish a strong allergic response followed by seven intragastric allergen challenges (Figure 1A), as previously described (29). In order to examine the potential role of SCF248 in the development and elicitation of food allergic responses we administered α-SCF248 mAb or control IgG by IP injection after the 4th intragastric challenge with ovalbumin (Figure 1A). Prior to injection the animals were assessed for clinical signs of food allergen responses by temperature drop and a standard anaphylaxis scale (0–5) as previously described (24). The data in Figure 1B indicate that mice in each of the groups, IgG and α-SCF248, had similar anaphylactic characteristics and based upon temperature loss and anaphylactic scale prior to therapeutic administration of antibody. The animals were then subjected to three additional challenges in the following week while being treated with α-SCF248 or control antibody. After the 7th challenge mice were assessed by temperature and anaphylactic scores for the severity of their responses. Those mice treated with α-SCF248 displayed a minimal loss of temperature and a minimal anaphylactic score overall, while those treated with control IgG showed a significant decrease in body temperature and a substantial anaphylactic response (Figure 1C).

**Figure 2:**
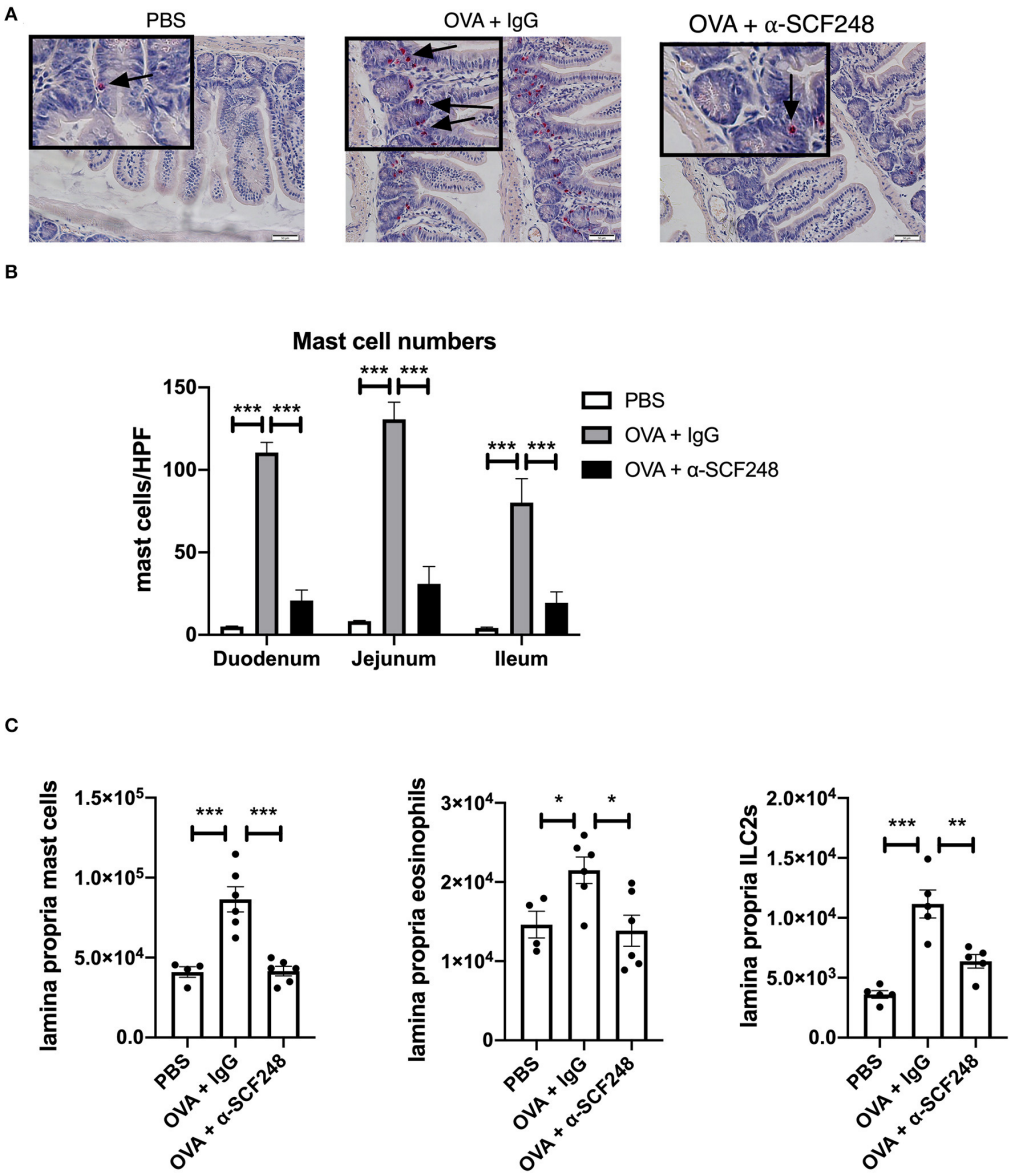
Anti-SCF248 treatment reduces mast cells, eosinophils, and ILC2s in the intestines of allergic mice. **(A)** Representative sections of the small intestine stained with chloroacetate esterase to visualize mast cells. Details are shown in the inset boxes. Scale bar = 50 μm. Mast cells are indicated by arrows. **(B)** Mast cells were counted in the duodenum, jejunum, and ileum. Numbers represent the mean of five high-powered fields per section. **(C)** Mast cells, eosinophils, and ILC2s in the small intestine lamina propria were quantified by flow cytometry. Results are from four to six mice per group and represent one of three independent experiments. *p < 0.05, **p < 0.01, ***p < 0.001.

To further examine whether this effect was associated with local mast cell numbers in the GI tract, histopathologic staining was utilized with non-specific esterase for identification of mast cell numbers (Figure 2). The histology shown in Figure 2A represent that while PBS treated, non-allergic mice have virtually no mast cells evident, ovalbumin sensitized and challenged mice have significant increases in esterase positive mast cells in the small intestine. Those animals treated with α-SCF248 appear to have a significant decrease in mast cell staining. Enumeration of mast cell numbers in stained slides demonstrate that there were significant increases in the control treated food allergic mice that were significantly reduced throughout the small intestine, the duodenum, jejunum, and ileum (Figure 2B). Mast cells in the lamina propria were also analyzed by flow cytometry following enzymatic digestion, and these results confirmed a decrease in the number of mast cells in mice treated with α-SCF248 (Figure 2C). Representative flow cytometry plots with gating strategies are shown in Supplementary Figure 1. We also noted a decrease in the number of eosinophils and type 2 innate lymphoid cells (ILC2), indicating an overall decrease in the T-helper type 2 (Th2) environment in the small intestine in the absence of SCF248 signaling.

### Neutralization of SCF248 Alters Th2 Cytokine Profiles

**Figure 3:**
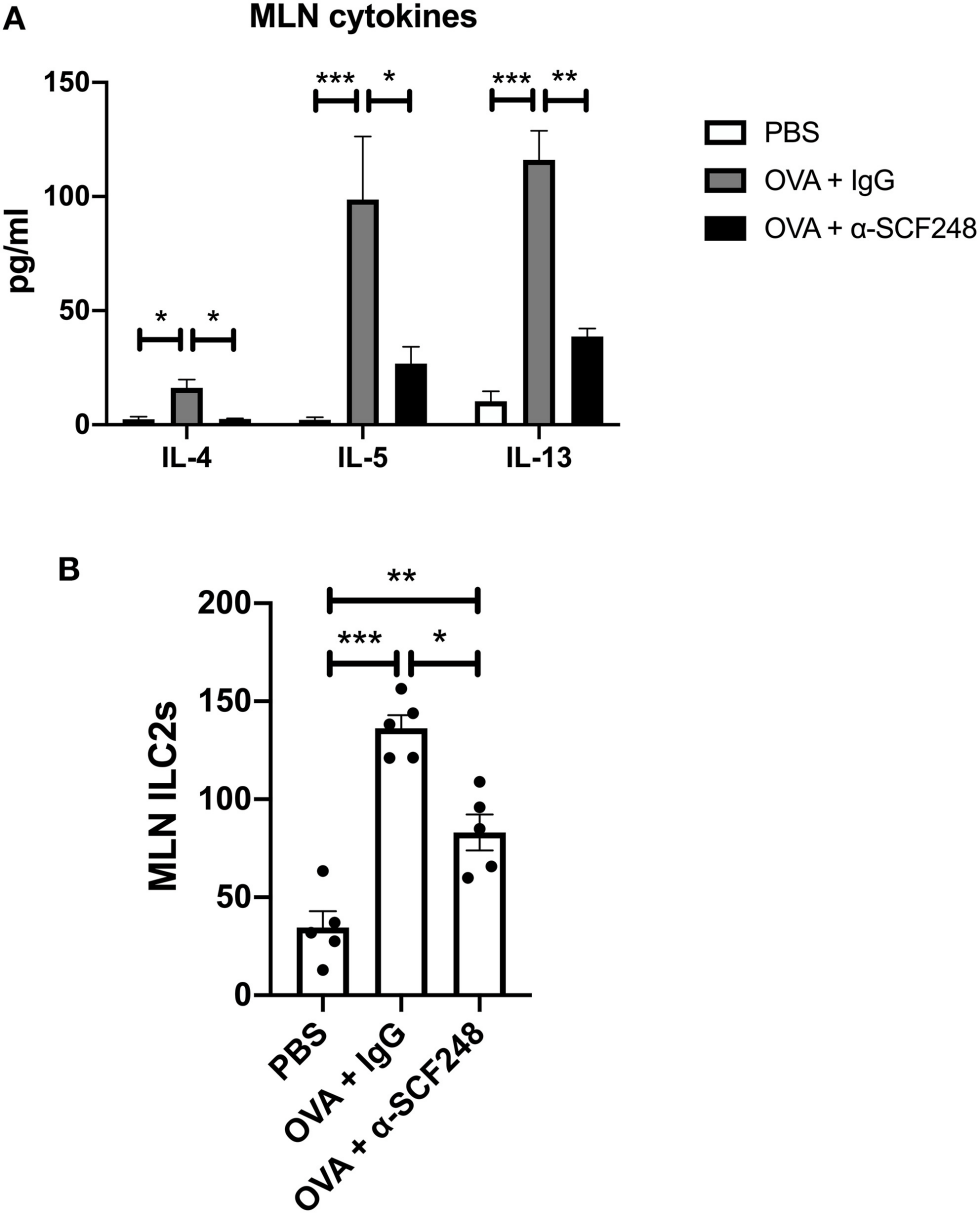
The Th2 response is decreased in the lymph nodes of mice treated with α-SCF248. **(A)** Mesenteric lymph nodes were removed and single cell suspensions were restimulated with OVA. Th2 cytokines were measured in the supernatant by Bioplex assay after 48 h of culture. **(B)** The number of ILC2s in the mesenteric lymph nodes were measured by flow cytometry. Five mice were used in each group, and data represent one of three independent experiments. *p < 0.05, **p < 0.01, ***p < 0.001.

In order to better understand whether blocking SCF248 alters the ongoing allergic responses and can change immune phenotypes mesenteric lymph nodes were isolated and restimulated with ovalbumin. The data presented in Figure 3A illustrate that inhibition of SCF248 during the challenge period in food allergic animals reduced type 2 cytokines, IL-4, IL-5, and IL-13, although there were no differences in the overall number of cells from the mesenteric lymph nodes. Our studies have previously identified SCF248 as an inducer of ILC2, as these are c-kit^+^ cell populations (22). When ILC2 numbers were examined in the mesenteric lymph nodes by flow cytometry, we also observed decreases in these cells that likely contribute to the type 2 allergic environment (Figure 3B), while there were no differences in Th2 cytokines in unstimulated cells from these animals (Supplementary Figure 2).

### Decreased Mast Cell Activity Following SCF248 Neutralization

**Figure 4:**
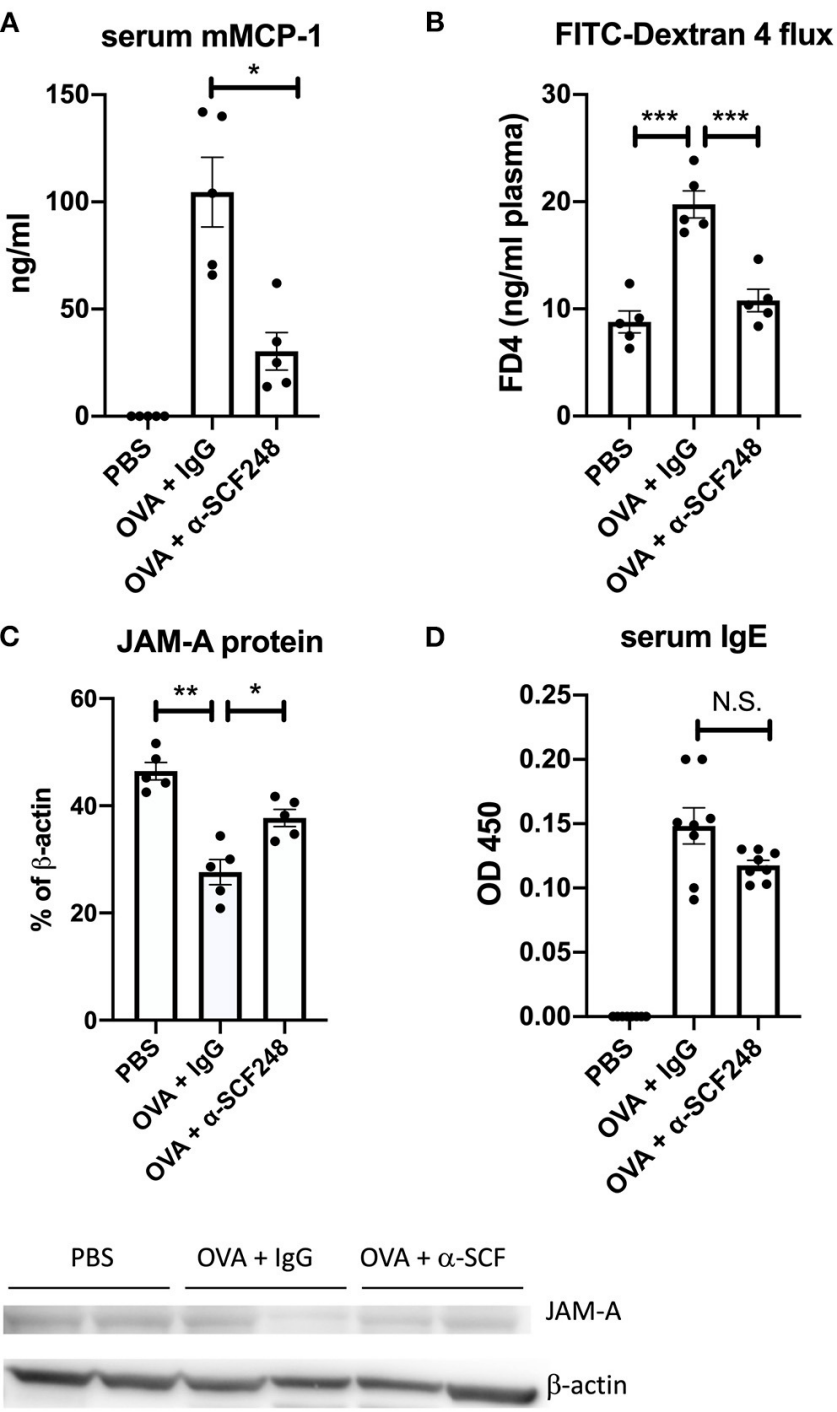
Mast cell activation and intestinal permeability are decreased in mice treated with α-SCF248. **(A)** Mast cell activation was assessed by measuring murine mast cell protease concentration in the serum 30 min after final challenge. Protease concentration was measured by ELISA. **(B)** Intestinal permeability was analyzed by delivering 4 kDa FITC-dextran by oral gavage, then measuring FITC in the plasma by fluorescence 4 h later. **(C)** Western blot of JAM-A was performed from the small intestine, with b-actin as a control. Densitometry analysis of JAM-A results are shown as a percentage of b-actin control. Each lane represents a different tissue sample, and one of two representative blots is shown. **(D)** OVA-specific IgE was measured in the serum of allergic mice by ELISA. Results represent one of three independent experiments with four to five mice per group. ELISA samples were run in duplicate, in addition to the biological replicates. *p < 0.05, **p < 0.01, ***p < 0.001.

When mast cells degranulate, one of the inflammatory mediators that is released is the specific protease murine mast cell protease-1 (mMCP1). We measured mMCP1 levels in the serum 30 min after challenge and found a significant reduction in the levels of this protease in the serum of α-SCF248 treated mice (Figure 4A). The activation of mast cells can affect the barrier function of the intestinal epithelium. To test this, mice were gavaged with 4 kD FITC-Dextran, and plasma was collected to measure the concentration of this molecule through the epithelial barrier. We found that mice treated with OVA had increased FITC-Dextran in the plasma, but this was inhibited in mice treated with α-SCF248 (Figure 4B), indicating that the decreased number and activation of mast cells protects barrier function. To support this, we performed a Western blot for JAM-A in the jejunum. JAM-A is part of the tight junction between epithelial cells in the small intestine, and contributes to barrier function (30). We found that JAM-A expression in the small intestine was decreased in OVA-treated mice, but that this was rescued by treating mice with α-SCF248 (Figure 4C, full blots shown in Supplementary Figure 3). Therefore, the increased number of mast cells correlates with decreased JAM-A in the GI tract, and an increase in intestinal permeability. In IgE-mediated food allergy, mast cells degranulation occurs when IgE bound to the mast cell encounters its antigen. We therefore measured OVA-specific IgE in the serum of allergic mice. We did not observe any difference in mice treated with control IgG or α-SCF248, indicating that there was no difference in sensitization to OVA (Figure 4D). There was no effect on other immunoglobulins, including IgG1, IgG2a, and IgG2b. Thus, the neutralization of SCF248 significantly reduced the anaphylactic responses and was correlated to the number and activation of mast cells in the gastrointestinal tract of allergic mice.

### Inhibition of Food Induced Anaphylaxis in Established Food Allergy Responses

**Figure 5:**
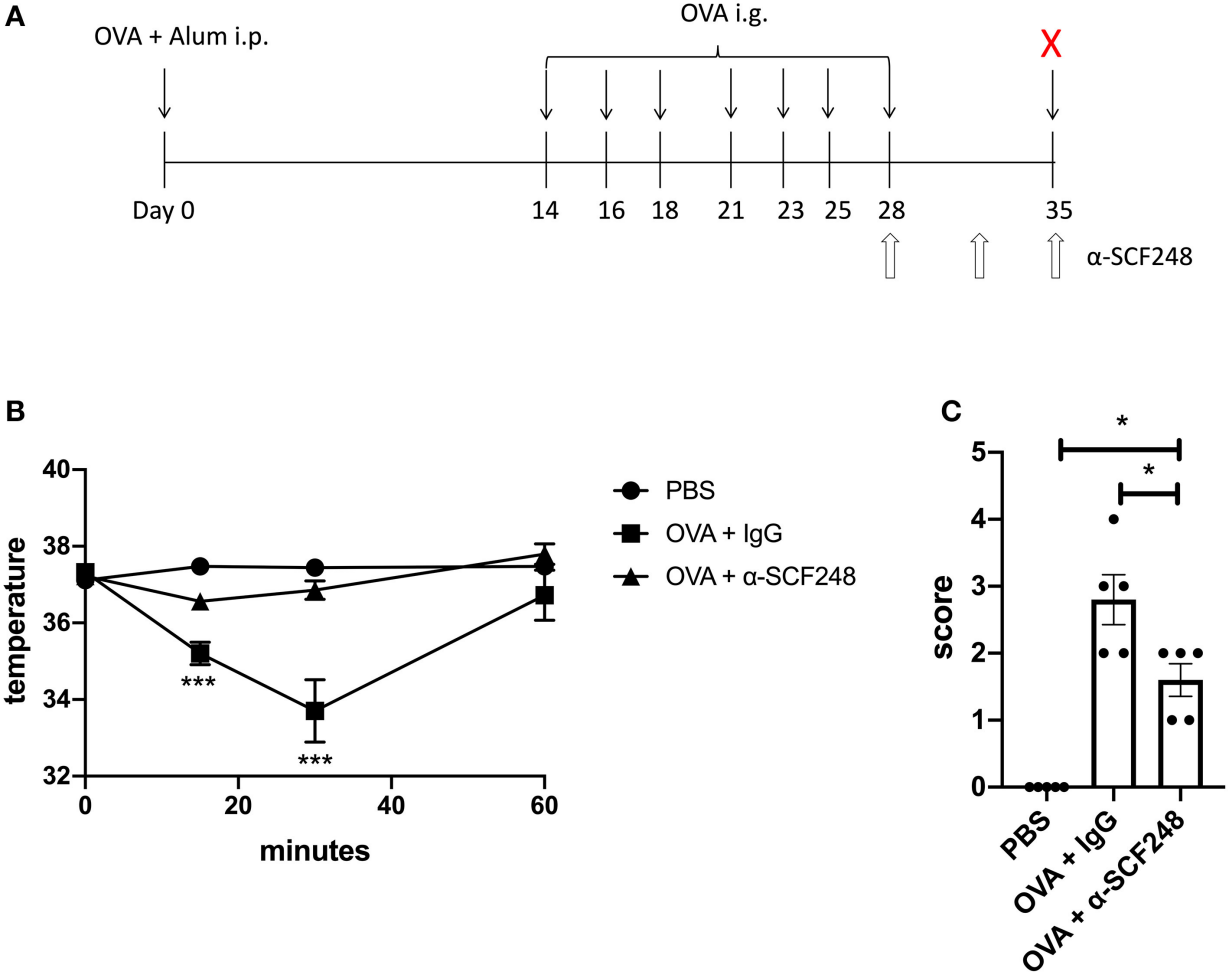
Anti-SCF248 treatment protects mice in a model of established food allergy. **(A)** Food allergy was induced by sensitizing mice with OVA and alum, followed by seven challenges with OVA, ending on day 28. SCF248 was blocked by injection with a monoclonal antibody on days 28, 31, and 35. Mice were given one additional challenge with OVA on day 35, following SCF248 neutralization. **(B)** Rectal temperatures were recorded for at 15, 30, and 60 min following challenge at day 35. **(C)** Mice were monitored for 60 min following final challenge, and assigned a score based on clinical symptoms. Results are from five mice per group and represent one of three independent experiments. *p < 0.05, ***p < 0.001.

The previous studies indicated that blocking SCF248 during ongoing food challenges reduces the severity of the food allergic responses. In order to better define whether SCF248 neutralization could inhibit the re-occurrence of an established response a second model was established. In this model (Figure 5A) the antibody was administered during a one week “rest” period where no food challenge was administered after the original model was established followed by a food challenge to the sensitized food allergic mice. The data presented in Figure 5 demonstrates that inhibition of SCF248 abrogates the effects of food induced anaphylaxis as indicated by no decrease in temperature in α-SCF248 treated animals in this more chronic model of established food allergy (Figure 5B). Similarly, the overall clinical parameters were reduced as indicated by the anaphylactic index (Figure 5C) that includes diarrhea and itching.

**Figure 6:**
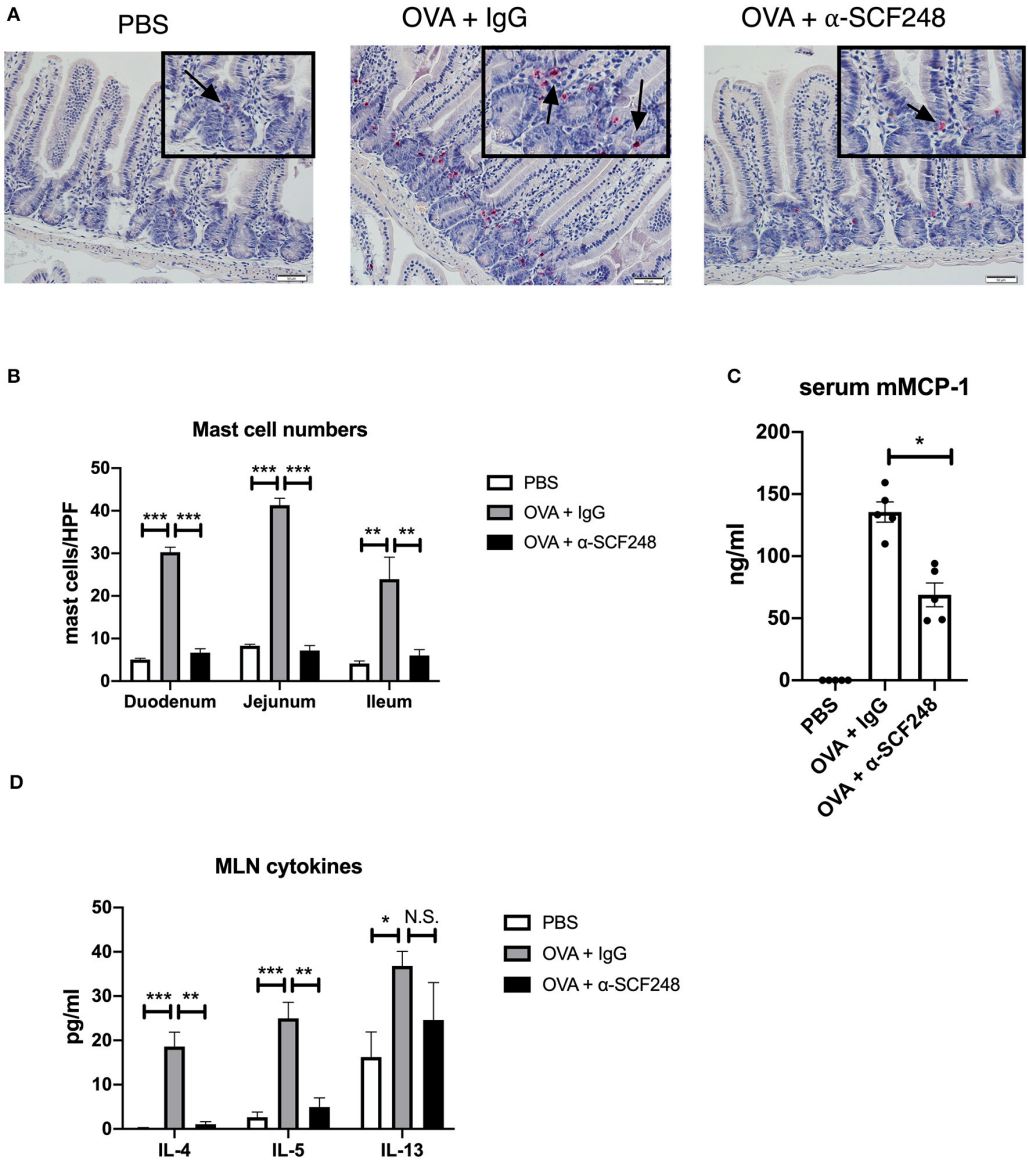
Anti-SCF248 treatment decreases intestinal mast cells and Th2 inflammation in model of established food allergy. **(A)** Mast cells were enumerated following staining of sections with chloroacetate esterase to visualize cells. Details are shown in the inset boxes. Scale bar = 50 μm. Arrows point to mast cells. **(B)** Mast cell numbers per high powered field were determined by counting five sections each in the duodenum, jejunum, and ileum. **(C)** Murine mast cell protease was measured by ELISA to determine the extent of mast cell activation. **(D)** Mesenteric lymph nodes were dissociated and single cell suspensions restimulated with OVA. After 48 h of culture, Th2 cytokines were measured in the supernatants by Bioplex assay. Five mice were used in each group, and data represent one of three independent experiments. *p < 0.05, **p < 0.01, ***p < 0.001.

We enumerated the mast cells in the small intestine by staining sections with chloroacetate esterase. As previously, we found that blocking SCF248 in an established model of food allergy resulted in decreased mast cell numbers in the tissue (Figures 6A,B). As a measure of the extent of mast cell degranulation, the serum mMCP-1 enzyme levels were significantly reduced in the α-SCF248 treated animals (Figure 6C). Finally, we measured the Th2 environment in these animals by restimulating mesenteric lymph node cells with OVA. We found that treating mice with α-SCF248 decreased IL-4 and IL-5 production, although there were no differences in IL-13 in allergic mice treated with α-SCF248 or control IgG (Figure 6D). Additionally, there were no differences in IFN-g or IL-17, indicating that only Th2 cytokines are altered by the neutralization of SCF248. Thus, inhibition of this pathway that is known to be involved in the expansion and activation of mast cells reduces the severity of food induced anaphylaxis.

## Discussion

The elicitation of severe allergic food responses has increased significantly over the past two decades and has caused changes in social and personal behavior in those households where it is present. The critical cellular mediator during a food anaphylactic response is the mast cell. A number of studies have suggested that the number of mast cells in the GI tract may correspond to the generation of anaphylaxis (31, 32). This is also likely accompanied by the ability of food to move through a normally tight epithelial barrier in the GI tract, which is modified by the local activation of mast cell mediated responses (33). Therefore, a central premise would be to control mast cell numbers in tissue that can provide the initial response to limit amplification in subsequent exposures due to the breakdown in barrier from the previous episode. The clinical observations regarding IgE levels and the ability to predict an anaphylactic food challenge at any given time is likely complicated by these factors, numbers of mast cells and barrier maintenance. The present studies demonstrate that by blocking SCF248 levels using a therapeutic antibody approach that a reduction in mast cells throughout the small intestine was achieved. This correlates well with genetic studies in mice that only express the SCF220 splice variant and not SCF248 that lack peripheral mast cells, even though myeloid bone marrow progenitor cell populations were not affected (21, 32). The reduction in clinical disease parameters in the food allergy challenges and the reduction of mast cells and type 2 cytokines also contributed to the protective phenotype after a therapeutic challenge in these studies. Thus, a reasonable strategy for reducing the incidence of food anaphylaxis would be to reduce mast cell numbers and therefore the anaphylactic environment would be mitigated. While we have not examined the effect of a-SCF248 antibody on steady state mast cell numbers, the genetic evidence suggests that tissue mast cells are dependent upon SCF248 and not SCF220 (23). Furthermore, the antibody used in this study does not recognized the cleaved domain of SCF248, and is therefore unlikely to interact with mast cells. Instead, the antibody targets the SCF248 isoform on fibroblasts, leading to a reduction in the amount of the cleaved domain available to bind to mast cells (23). However, the long-term effects of a-SCF248 should still be a consideration for future studies, particularly with regards to the possibility of using this antibody in a clinical setting.

One outcome in these studies was the reduction in mast cell numbers and activation, combined with reduced gut leak. These results suggest an important interaction between the systemic anaphylactic responses and the ability of allergen to pass through the gut epithelium and activate the immune cells in the lamina propria. In conjunction with these observations was the reduced numbers of Type 2-associated immune cells locally in the tissue as well as a reduced recall response from draining lymph node cultures. Similar studies using a peanut allergy model of anaphylaxis in mice in which mast cells were targeted found similar changes in the local Th2 response from the draining lymph nodes (34). Thus, these data suggest that reducing local gut mast numbers and local activation, mediated by IgE, may reduce the barrier leak of food antigens into the periphery and alter future responses, including protection from anaphylactic responses. This central premise may help to explain differences in how patients with systemic responses to food do or do not respond to a food challenge.

Epithelial barriers are established with multiple protein interactions that include claudins, occludins, junctional adhesion molecules and others that are differentially regulated to control movement of soluble antigens into the tissue and systemically (35–37). The critical role of these molecules has been established in models of inflammatory bowel diseases, but little is known about how their maintenance is associated with antigen sensitization (38). While more is known about barrier function and tolerance in food allergy, as well as the role of increased gastric acid production, the role of immune cell mediators in barrier function is not at well understood (39). Mast cells are the critical mediators of food allergy, but in order to trigger a reaction, antigen must bind to IgE on the mast cell in the lamina propria, which rapidly release a number of preformed mediators. Thus, food antigens must pass through the epithelial barrier. Mast cell tryptase, one of these mediators, has been shown to directly impact the epithelial barrier by decreasing junction adhesion molecule-A (JAM-A) (40). JAM-A is a critical protein in the tight junction, which is part of the mechanism that determines epithelial permeability. Previous studies have shown that mice that lack JAM-A in the intestinal epithelium have a leaky barrier, as determined by passage of 4 kDa FITC-dextran into the blood following oral gavage (30). Other studies have found that mast cell activation impacts epithelial barrier function in the small intestine. These include *in vitro* studies have shown that mast cell chymase can affect the distribution of the tight junction proteins ZO-1 and occludin, and can reduce the expression of the claudin five protein (41, 42). A number of cytokines produced by mast cells also affect barrier integrity, including TNF-a, IL-1b, and IL-9 (31, 43–45). In this study, we show that increased mast cell activation results in greater passage of FITC-dextran across the barrier in allergic mice, suggesting that increased permeability to OVA would result in more severe anaphylactic symptoms. However, mast cell activation, as measured by murine mast cell protease-1, is decreased in mice treated with SCF248 neutralizing antibody, although it must be noted that we did not directly visualize mast cell degranulation in the lamina propria. Nevertheless, the decrease in local mast cell numbers and systemic detection mast cell protease-1 correlates with increased barrier function and decreased disease.

In a food allergic reaction, and individual is sensitized to food antigen, resulting in an established Th2 immune response. This is sustained not only by Th2 cells, but also by type 2 innate lymphoid cells in the lamina propria of the small intestine, as well as in the local lymph nodes (46–49). As ILC2s express c-kit, the receptor for SCF, neutralizing SCF248 has the potential to decrease the Th2 environment prior to allergic challenge. Indeed, we found that the number of ILC2s in the lamina propria as well as in the mesenteric lymph nodes were lower in mice treated with α-SCF248 compared to allergic mice treated with control IgG. Previous studies have found that increased mast cell numbers drive ILC2 expansion (50). Here, we find that both ILC2 numbers and mast cell numbers are decreased in mice treated with α-SCF248, however whether the decrease in ILC2 numbers is due to the lack of c-kit signaling on the cell or is linked to mast cell number is currently unknown. In addition, we found that the overall Th2 response was decreased, including the antigen specific T cell reaction in the lymph nodes, indicating that SCF248 is important for maintaining the Th2 environment in an allergic individual. This is further supported by the decreased number of eosinophils in the lamina propria of mice treated with α-SCF248 antibody.

In this study, we examined the role of SCF248 in sensitized animals. In some experiments, we began challenging the mice, and when they began to show symptoms, we neutralized SCF248. In this model, each subsequent challenge results in increased severity of anaphylactic symptoms, as mast cells continue to accumulate with each challenge. Therefore, we are able to halt the increase in mast cells following the initial challenges. Because all animals were sensitized equally, mice treated with α-SCF248 maintain similar levels of OVA-specific IgE compared to mice treated with control IgG. However, with fewer mast cells to bind to, this IgE is unable to activate a strong anaphylactic response. These results were maintained even in a delayed challenge model, in which SCF248 was neutralized following a full allergic response. Together, our findings suggest that SCF248 is an attractive target to minimize robust anaphylaxis in established food allergy.

## Data Availability Statement

The raw data supporting the conclusions of this article will be made available by the authors, without undue reservation.

## Ethics Statement

The animal study was reviewed and approved by University of Michigan Institutional Animal Care and Use Committee.

## Author Contributions

CP and NWL conceived of the project and wrote the manuscript. CP, AJR, and WF performed the experiments and analyzed the data. All authors contributed to the article and approved the submitted version.

## Conflict of Interest

NL was a co-founder of Opsidio, LLC, which is developing anti-SCF248 for commercial use. The remaining authors declare that the research was conducted in the absence of any commercial or financial relationships that could be construed as a potential conflict of interest.

## Supplementary Material

Supplementary Figure 1 Representative flow plots for cellular analysis. **(A)** Gating strategy for ILC2s. **(B)** Gating for mast cells, gated on CD45^+^ cells. **(C)** Gating for eosinophils.

Supplementary Figure 2 Baseline Th2 cytokines from lymph nodes are unchanged in OVA-treated mice compared to control. Mesenteric lymph nodes were dissociated and single cells suspensions were cultured without restimulation. Bioplex analysis was performed after 48 h.

Supplementary Figure 3 JAM-A Western blot. Detection of JAM-A antibody, followed by stripping of the blot and reprobing with b-actin.
