# Supplementary figures and images for: Stem Cell Factor Neutralization Protects From Severe Anaphylaxis in a Murine Model of Food Allergy
Source: Front Immunol. 2021 Mar 9;12:604192. doi: 10.3389/fimmu.2021.604192 (PMC8005333; doi:10.3389/fimmu.2021.604192)

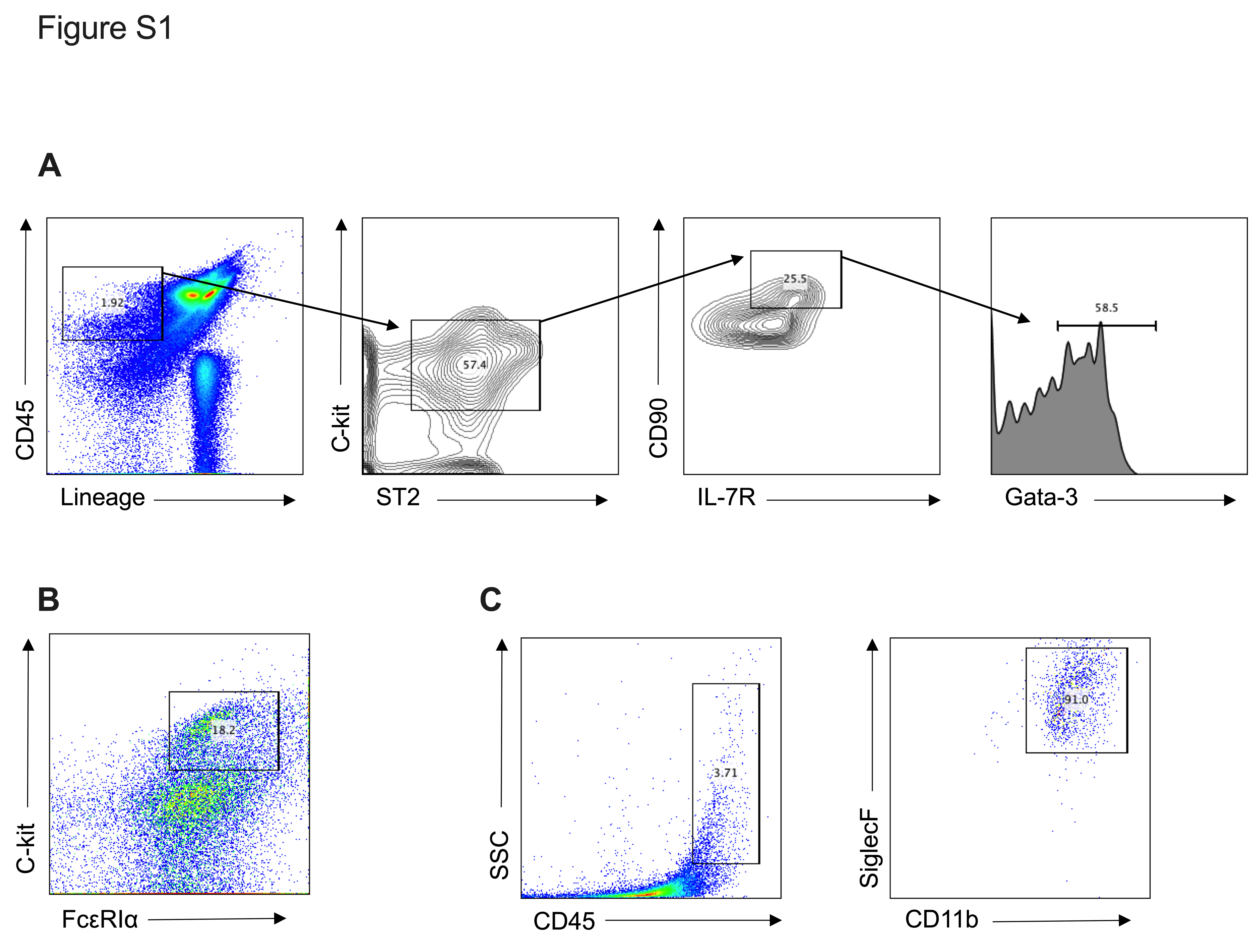

Supplement: Supplementary Figure 1 — Representative flow plots for cellular analysis. (A) Gating strategy for ILC2s. (B) Gating for mast cells, gated on CD45+ cells. (C) Gating for eosinophils. [file Image_1.TIFF]

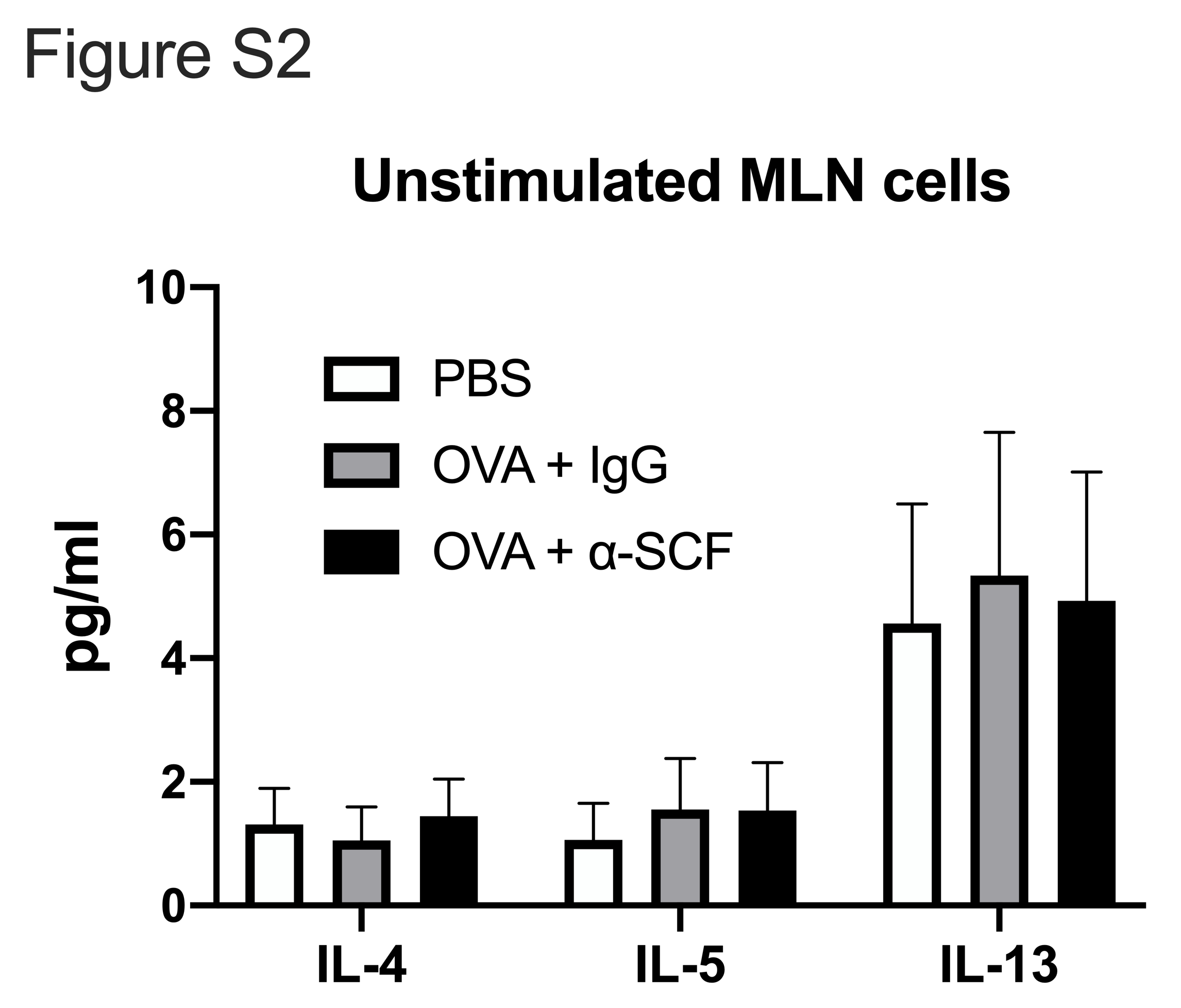

Supplement: Supplementary Figure 2 — Baseline Th2 cytokines from lymph nodes are unchanged in OVA-treated mice compared to control. Mesenteric lymph nodes were dissociated and single cells suspensions were cultured without restimulation. Bioplex analysis was performed after 48 h. [file Image_2.TIFF]

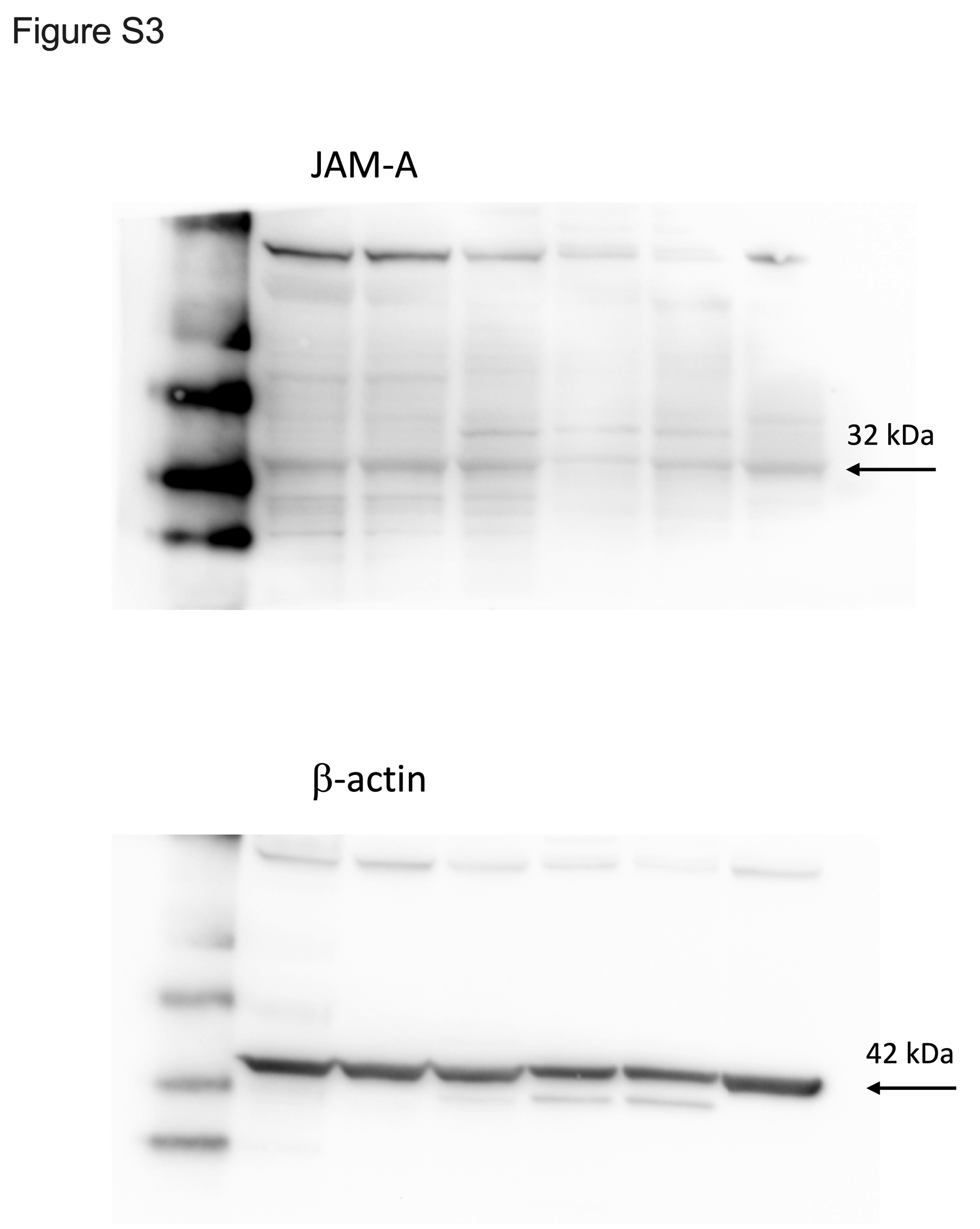

Supplement: Supplementary Figure 3 — JAM-A Western blot. Detection of JAM-A antibody, followed by stripping of the blot and reprobing with b-actin. [file Image_3.TIFF]
